# Radiation therapy for a case of poikilodermatous plaques in an otherwise healthy young man: A case report

**DOI:** 10.1177/2050313X241274837

**Published:** 2024-10-09

**Authors:** Ursula Biba, McKayla J Poppens, Erin K Collier, Kyle Cheng

**Affiliations:** 1 David Geffen School of Medicine, University of California, Los Angeles, Los Angeles, CA, USA; 2 Division of Dermatology, Department of Medicine, David Geffen School of Medicine, University of California Los Angeles Health, Los Angeles, CA, USA

**Keywords:** Poikilodermatous, mycosis fungoides, T-cell, cutaneous lymphoma, radiation

## Abstract

Poikilodermatous mycosis fungoides is a rare variant of cutaneous T-cell lymphoma that is often misdiagnosed given its diverse clinical presentation. Often diagnosed as vitiligo or morphea, poikilodermatous mycosis fungoides can be asymptomatic or present as pruritic lesions. Discrepant signs and symptoms can lead to diagnostic delays. No consensus on its treatment currently exists, but treatment options include corticosteroids, phototherapy, and radiation. Here, we present a case of poikilodermatous mycosis fungoides in an otherwise healthy young man who showed limited improvement after years of treatment with topical antifungals, topical steroids, and phototherapy. Improvement was seen following a single session of radiation therapy, highlighting radiation’s potential in cases resistant to traditional first-line treatments. We propose that radiation may be efficacious for the treatment of poikilodermatous mycosis fungoides in cases of delayed diagnosis or resistance, and further research is needed to investigate radiation monotherapy as a treatment option for poikilodermatous mycosis fungoides.

## Introduction

Mycosis fungoides (MF) comprises 50%–80% of malignant cutaneous T-cell lymphomas, with varying clinical and histopathologic subtypes.^1,2^ Poikilodermatous mycosis fungoides (PMF) is a rare MF variant often misdiagnosed as vitiligo, morphea, lichen planus, or contact dermatitis.^1,3,4^ In prior case reports, PMF has presented as erythematous localized lesions, large plaques in flexural or bathing suit distributions, or papules that were asymptomatic or dry, burning, and pruritic.^1–3^ Challenges in recognizing these various presentations have led to diagnostic delays ranging from 31 months to 18 years.^1^ No consensus currently exists on the management of PMF, but treatment options include topicals (i.e., corticosteroids, bexarotene, carmustine, mechlorethamine, and imiquimod), phototherapy (psoralen, ultraviolet A, and narrow-band ultraviolet B [nbUVB]), and radiation.^2,4,5^ We propose that radiation may be efficacious for the treatment of PMF in cases of delayed diagnosis or resistance to first-line treatments.

## Case presentation

A 33-year-old previously healthy Hispanic male presented to dermatology for nonpruritic rashes on his right foot, right thigh, and left hip. The patient first noticed a rash on his right foot 20 years prior when he sought care elsewhere. A punch biopsy at that time revealed lichenoid dermatitis. Over the years, he trialed localized phototherapy, topical antifungals, and topical steroids with limited effect. In 2020, he noticed new rashes on his right inner thigh and left hip, prompting re-presentation in January 2022. At the time, he was asymptomatic and denied constitutional symptoms.

**Figure 1.**
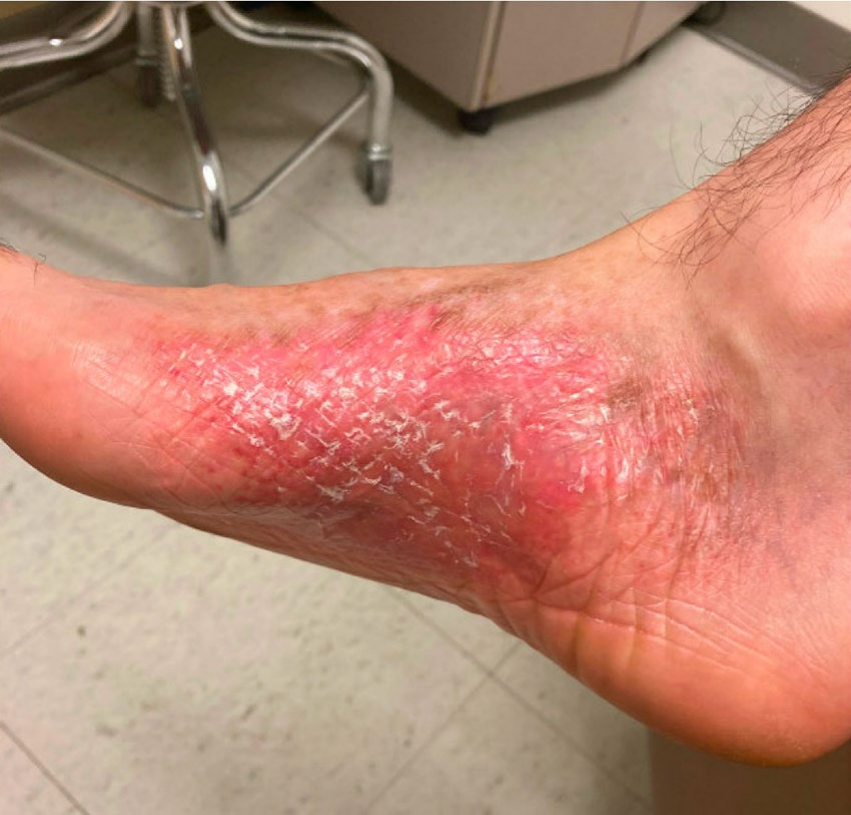
On the right medial foot is an ill-defined erythematous plaque with central scaling. Patient presented with history of this lesion for approximately 20 years. Image was taken following biopsy that confirmed diagnosis of poikilodermatous mycosis fungoides, prior to any radiation, UV, or topical therapy provided at UCLA. Treatments trialed up to this point of presentation included nbUVB, corticosteroids, and antifungals at an outside institution intermittently over many years per patient report. nbUVB: narrow-band ultraviolet B; UV: ultraviolet.

**Figure 2.**
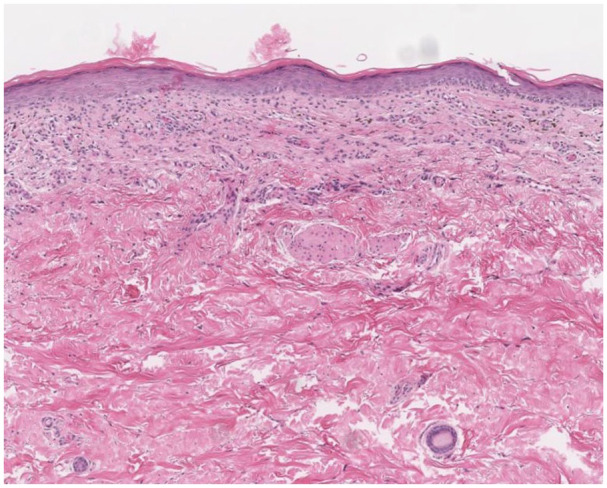
Punch biopsy of lesion showing a superficial lichenoid band of lymphocytes along the dermoepidermal junction with extension into epidermis. Hematoxylin and eosin stain, 10× magnification.

Physical examination revealed an erythematous scaly plaque on the right medial foot (Figure 1) and erythematous reticular atrophic plaques on the right medial thigh and left hip. Laboratory results were within normal limits. Infectious workup, flow cytometry, and peripheral blood smear were unrevealing. Punch biopsy of the three rashes revealed similar findings, including a superficial lichenoid band of lymphocytes along the dermoepidermal junction with extension into the overlying epidermis (Figure 2). Lesional lymphocytes showed enlargement and nuclear irregularity. Immunohistochemistry demonstrated strong CD3 and CD8 positivity, CD30 positivity in scattered enlarged lymphocytes, weak CD4 and CD2 positivity in a small subset of cells, CD5 negativity, CD7 negativity, and CD20 negativity. T-cell gene rearrangement studies showed clonal T-cell gene rearrangement for the *TRG* locus. Ultimately, the workup was consistent with Stage 1A CD8^+^ MF, poikilodermatous variant.

**Figure 3.**
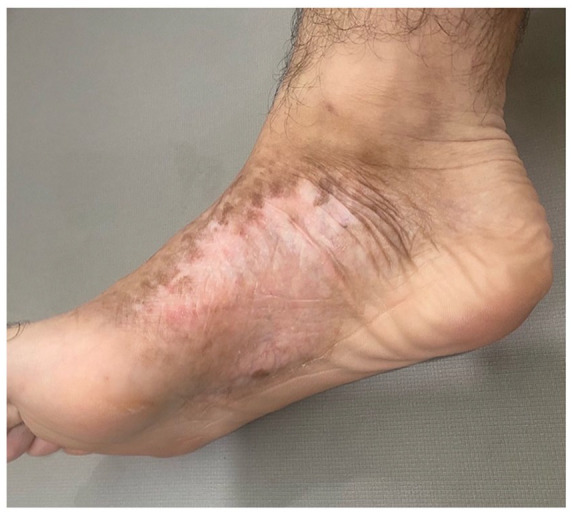
At 10-month follow-up after treatment with intensity-modulated radiation therapy (24 Gy/12 fractions), a residual hypopigmented patch is visible on the right medial foot. Treatment has also been supplemented with daily topical imiquimod application. Prior to radiation treatment, patient had trialed 5 months of nbUVB and topical clobetasol, tacrolimus, and mechlorethamine with minimal results. nbUVB: narrow-band ultraviolet B.

In the months after diagnosis, the patient failed several first-line treatments, including 5 months of nbUVB phototherapy and topicals, including clobetasol, tacrolimus, and mechlorethamine. The patient underwent intensity-modulated radiation therapy (24 Gy/12 fractions) to the foot lesion, with remarkable improvement in appearance and no evidence of recurrence 10 months post-treatment (Figure 3). The hip and inner thigh lesions began to fade with daily topical imiquimod and continued to show improvement at the time of the patient’s most recent visit 10 months after imiquimod initiation.

## Discussion

PMF often presents during the fourth or fifth decades of life, with symptom duration ranging from 2 to 10 years before diagnosis, and a 91-month median time to diagnosis.^6,7^ Of the MF subtypes, PMF has the longest delay to diagnosis.^8^ Despite this delay, PMF has presented with a 100% 5-year survival, which is longer than in other variants.^3,9^

PMF treatment resembles that of classic MF. Topical therapies include corticosteroids, bexarotene, carmustine, mechlorethamine, and psoralen, as well as phototherapy and radiation, which can be combined with systemic therapies, including retinoids or interferon-alpha.^2,4^ Some cases treated with topical steroids demonstrated improvements in symptoms and rash appearance, while others revealed minimal or no response.^3,4^ Topical bexarotene is not as well-tolerated and has not demonstrated efficacy as first-line treatment, but may stabilize lesions after treatment with phototherapy.^3,4^

Some cases recalcitrant to topical therapy improved after treatment with methotrexate, oral retinoids, or immunotherapy.^3^ Regarding phototherapy, treatment with psoralen and ultraviolet light A (PUVA) has demonstrated benefit in early disease stages.^6,7^ NbUVB therapy in combination with corticosteroids or acitretin has also shown moderate efficacy, but not without complications.^3^ In our case, our patient’s foot rash resolved following a single session of radiation therapy after limited improvement with topical medications and nbUVB therapy.

While treatment with PUVA or nbUVB is common in PMF management, electron beam radiation has also shown efficacy in recalcitrant MF. A retrospective study found that low-dose radiation (8 Gy/1 fraction) to localized MF led to a 93% response rate at 25 months without evidence of recurrence at follow-up.^10^ A case report supported the use of low-dose radiation for localized MF after the patient failed many topical and systemic treatments and phototherapy.^11^ Other studies of higher radiation doses showed approximately 90% disease-free survival after 20 Gy/10 fraction radiation and a 100% response rate after 30.6 Gy/1.8–2 fraction radiation.^10^ Another retrospective study showed similar results: MF lesions treated with a median dose of 25 Gy radiation therapy resulted in a 95% remission rate at 1-month and a 63% cure rate at 5-year follow-up.^12^

While the benefit of radiation therapy is limited to localized disease, total skin electron beam therapy (TSEBT) is also used when involvement is more widespread.^10^ A prospective study of MF patients found that low-dose TSEBT (12 Gy/12 fractions) led to clinical benefit in 21 months.^13^ A multicenter observational study of patients with MF or Sézary Syndrome also found an 89% response rate to TSEBT (8 Gy/2 fraction).^14^ Interestingly, a retrospective study comparing TSEBT at different radiation levels (12 Gy vs 36–40 Gy) in MF patients showed longer time without disease progression in the 36–40 Gy group (15.7 months vs 5.3 months).^15^

This case highlights the potential efficacy of radiation therapy in PMF that is unresponsive to standard MF treatments. Beyond other studies of radiation therapy in PMF, potential benefits can be explored based on studies of its use in other cutaneous T-cell lymphomas. Further research is needed to investigate the efficacy of radiation monotherapy for PMF, though it holds promise for an otherwise difficult to treat condition.
